# Social Determinants of Health ICD-10 Code Use by a Large Integrated Healthcare System

**DOI:** 10.3390/healthcare13212710

**Published:** 2025-10-27

**Authors:** Cynthia Hau, Janet M. Grubber, Ryan E. Ferguson, William C. Cushman, Areef Ishani, Peter A. Glassman, Colleen A. Hynes, Sarah M. Leatherman

**Affiliations:** 1 Cooperative Studies Program Coordinating Center, VA Boston Healthcare System, Boston, MA 02111, USA; 2 Department of Medicine, Boston University Chobanian & Avedisian School of Medicine, Boston, MA 02118, USA; 3 Medical Service, Memphis VA Medical Center, Memphis, TN 38105, USA; 4 Department of Preventive Medicine, University of Tennessee Health Science Center, Memphis, TN 38163, USA; 5 Minneapolis VA Healthcare System, Minneapolis, MN 55417, USA; 6 Department of Medicine, University of Minnesota, Minneapolis, MN 55455, USA; 7 Pharmacy Benefits Management Services, Department of Veterans Affairs, Washington, DC 20420, USA; 8 VA Greater Los Angeles Healthcare System, Los Angeles, CA 90073, USA; 9 David Geffen School of Medicine, University of California, Los Angeles, CA 90095, USA; 10 Department of Biostatistics, School of Public Health, Boston University, Boston, MA 02118, USA

**Keywords:** social determinants of health, SDOH ICD record, code utilization, EHR documentation

## Abstract

**Background/Objectives**: Identifying social determinants of health (SDOH) is important for effective clinical care. The ICD-10 introduced diagnostic categories to describe patients’ adverse SDOH, but these codes are infrequently used across health systems, presenting challenges to implement data-driven healthcare. This study illustrates SDOH code utilization within a setting that is recognized as one of the largest integrated healthcare systems across the United States. **Methods**: Real-world clinical data were used with ICD-10 SDOH records obtained from 13,523 participants randomized into the Diuretic Comparison Project, a pragmatic trial conducted within the Veterans Affairs (VA) Health Care System between 2016 and 2022. SDOH code utilization was assessed across study years and among the specialized outpatient clinics. **Results**: A total of 29,305 SDOH records were identified, and 99.2% were from outpatient encounters. Social, mental, and housing care services generated the most SDOH records. Moreover, 3894 (28.8%) participants had at least one SDOH record during the 6-year period. Particular, 6.9% of participants had a record in the first year, and this increased to 7.6%, 8.1%, 8.7%, 9.6%, 10.3% in consecutive years. **Conclusions**: Our results suggest that SDOH code utilization has continued to improve within the VA, but SDOH assessments may not occur annually or be performed systematically within an integrated health setting. Much work is needed to develop universal screening tools and mandate routine SDOH evaluations. Nevertheless, a persistent increase in the counts of ICD-10 SDOH records shows a positive movement towards systematic documentation, supporting service providers to efficiently identify patients with adverse SDOH.

## 1. Introduction

Addressing health inequalities is a clinical priority and a key objective for Healthy People 2020 and 2030 [1,2]. Social determinants affecting health outcomes, such as inadequate income or poor education, causing delays in care, are increasingly recognized by clinical service providers [3]. Social determinants of health (SDOH), including education, socioeconomics, employment, and living status, have a strong relationship with mental, physical, and emotional well-being [4]. These factors may influence access to healthy choices and quality care, demonstrating high associations with clinical outcomes [5,6].

Identifying and reporting adverse SDOH can help service providers minimize the negative effects on patient health through effective clinical interventions [7]. For instance, electronic health records (EHR) revealing patients with housing or economic difficulties may indicate potential problems with digital literacy or technology access, making the use of telehealth appointments challenging. This enables providers to limit tele-visits among affected patients, preventing exacerbations of existing health inequalities, especially when routine care is shifting towards remote delivery and digital monitoring [8]. Recently, incorporating SDOH data is recommended for precision medicine, an emerging clinical intervention that applies multifaceted EHR data (including genomic biomarkers and medical history) to inform treatment decisions. A concern with this approach is the application of EHR data reiterates access inequities and may contain less information on patients from underserved areas or disadvantaged backgrounds. Consequently, to reach its full potential, SDOH data should be considered in designing a well-suited and deliverable clinical intervention [9].

There is a growing interest in collecting SDOH data [10]. The 10th International Classification of Disease (ICD-10) released in 2015 has provided a reporting mechanism, with structured diagnosis codes describing problems related to social circumstances, including education/literacy (Z55), employment (Z56), occupational exposures (Z57), physical environment (Z58), housing/economic (Z59), social environment (Z60–Z63), and psychosocial conditions (Z64–Z65). In 2024, the Centers of Medicare and Medicaid Services (CMS) implemented reimbursement strategies to promote social risk assessments and electronic data capture during inpatient care [11]. Despite continuous efforts to promote SDOH evaluation, overall use of the SDOH ICD-10 codes remains low across healthcare systems [12,13,14,15], with less than 2% of CMS beneficiaries having an SDOH record in 2017 and 2020 [16,17].

Within the VA, a regional healthcare system has initiated SDOH screening implementation, named the Assessing Circumstances and Offering Resources for Needs (ACORN) project [18]. Through a self-reported process and among the 268 patients who completed the screenings, about 50% identified as having health-related social needs. However, it remains uncertain whether the percentage of VA patients with ICD-10 SDOH records is comparable to the ACORN results. This study describes SDOH code use within the VA Health Care System, examining SDOH record availability among patients from a health setting that emphasizes risk assessments and care coordination. Embedding social workers in primary care teams and expanding telehealth services are some integrated care models instigated by the VA. Although this analysis, based on ICD-10 data, cannot indicate the frequency of screening performed by providers, the record counts can improve our understanding of SDOH record availability within an integrated healthcare setting.

## 2. Materials and Methods

### 2.1. Diuretic Comparison Project (DCP)

Real-world clinical data were obtained from participants of the Diuretic Comparison Project (DCP), a pragmatic trial embedded within the Veterans Affairs (VA) Health Care System to compare two antihypertensive diuretics for prevention of cardiovascular (CV) events [19,20]. DCP provided a great opportunity to assess SDOH code utilization because: (1) the VA has one of the largest integrated health networks that advocates care coordination across different providers, improving continuity as compared to other settings with a fragmented care model; (2) it randomized a nationally representative sample of older VA patients with a decent portion of participants from rural and lower socioeconomic backgrounds [21]; and (3) the DCP study sample may provide fertile ground for the use of SDOH codes, as the relationship between SDOH and CV health is known in that economic disadvantage is frequently linked to poor CV outcomes and post-cardiac care [22]. DCP was registered under the Clinical Trials Registry (NCT02185417) and approved by the VA Central Institutional Review Board. Complete trial design and primary study results have been described elsewhere [19,20]. Briefly, all DCP activities were performed in tandem with VA’s usual clinical practice, including consent, randomization, safety surveillance, outcome ascertainment, and trial follow-up. Eligible patients were enrolled from the 72 participating VA medical centers that encompassed over 500 primary care clinics located across the US and Puerto Rico. Provider assent was obtained prior to randomizing each eligible patient. Verbal informed consent was collected for those who successfully completed the EHR-based trial enrollment.

### 2.2. Data Collection

This secondary analysis was performed using data collected from 13,523 participants randomized into DCP. Although there was an SDOH screening initiative within a regional setting [18], there were no universal screening tools or standardized clinical workflows implemented across the VA. This study focused on examining SDOH code utilization with all Z-code records included for analysis, enabling the computation of total records during the DCP study period (June 2016 and May 2022).

Inpatient and outpatient records were obtained from the VA Corporate Data Warehouse (CDW), a national data repository of EHR data for all VA patients [23]. ICD-10 codes referring to health problems associated with socioeconomic or psychosocial circumstances were selected, ranging from Z55–Z65, with the exclusion of Z61 due to it being relevant to pediatric patients only. Z58 was introduced during mid-study (starting October 2021). The analysis was planned to include Z58 records for respective years, and the total record frequencies included multiple Z-codes associated with single encounters. For outpatient data, primary and secondary clinic types (i.e., clinic stop codes) were also extracted. The 3-digit clinic stop codes are used within the VA to classify various healthcare services provided to patients. There is a coding scheme to identify the primary or secondary providers, with clinics flagged as ‘primary’ to classify providers who initiated the healthcare appointment, whereas ‘secondary’ indicates additional healthcare services provided during the same appointment. Code usage frequencies were assessed to provide a fundamental understanding of SDOH record availability within the VA Health Care System. Participants were classified as having a Z-code (SDOH-present) and not having any (SDOH-absent) during the 6-year study period. These were used as a real-world clinical measure indicating the proportions of patients for whom adverse SDOH risks were reported at the point of care. Baseline socio-demographic factors (age, sex, race, ethnicity, rurality, smoking status), physiological measures (systolic blood pressure, body mass index), and medical comorbidities (history of diabetes, heart failure, myocardial infarction, stroke, kidney disease) were described among the SDOH groups. While some of the baseline characteristics are often included as SDOH variables, the categorization of SDOH-present and -absent in the current manuscript was based on the existence of an ICD-10 record in EHR. Systolic blood pressure and body mass index were included as overall health indicators around the baseline timeframe, in addition to the review of prior medical history.

### 2.3. Statistical Analyses

Patient characteristics were descriptively compared between the SDOH groups. Continuous variables were reported as means ± standard deviations (SD) or medians with interquartile ranges (IQR) for non-Gaussian distributed variables. Discrete or categorical variables were reported using frequencies and percentages.

Subcodes within the major SDOH Z-codes were combined to assess utilization of the broader categories (Appendix A). Coding frequencies were computed for each major Z-code, both overall and for each study year. Corresponding record counts included all visits with ICD-10 SDOH codes listed during a given year, which was defined according to the DCP study initiation (starting on June 1st of one year and ending on May 31st of the following year). The percentage of participants having an SDOH record was calculated as those who received ≥1 code within a study year, divided by the proportion of patients alive and not withdrawn at the start of that year.

SDOH records generated within a specialized outpatient service were grouped based on the associated clinic stop codes. Specialty services with the highest coding frequencies were reported. These are shown as an overall use, regardless of the primary/secondary provider code assignment, and separately for a more comprehensive review (Supplemental Tables S1 and S2). The proportion of participants having multiple SDOH diagnoses was also examined, determined as the number of distinct years in which patients had >1 recorded Z-code among the SDOH-present group. All analyses were conducted using SAS software version 9.4 (SAS Institute, Cary, NC, USA).

## 3. Results

A total of 29,305 SDOH codes were identified across 13,523 randomized participants during the study period, and 99.2% were from outpatient encounters (with 96–99% of participants having an outpatient visit during each study year). Moreover, 3894 (28.8%) participants were categorized as SDOH-present and 9629 (71.2%) as SDOH-absent. Compared to the SDOH-absent group, the SDOH-present group had more participants as females, Black/African American, Hispanic/Latino, urban residents, current smokers, and those with chronic conditions (Table 1). Baseline age, body mass index, and systolic blood pressure were similar between groups.

### 3.1. SDOH Coding Frequency

**Figure 1:**
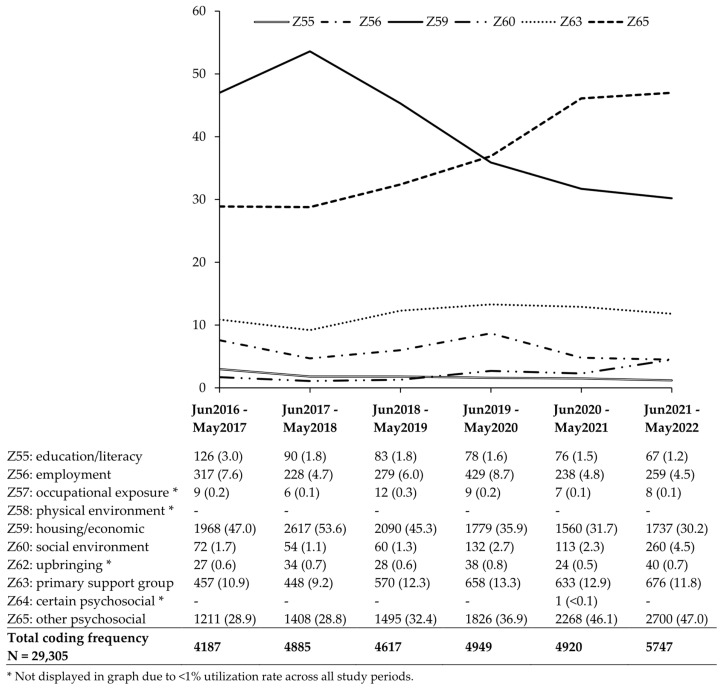
Social determinants of health (SDOH) Z-code utilization, June 2016 through May 2022.

The data presented in Figure 1 reveal an overall increase in the use of SDOH codes. Z59 (housing and economic issues; n = 11,751; 40.4% of total records) and Z65 (problems with “other” psychosocial circumstances; n = 10,908; 37.5% of total records) were coded most frequently. Observations of Z65 were contrary to the recording of Z64 (problems with “certain” psychosocial circumstances), which occurred only once. Z58 (problems with the physical environment) was never reported during the entire period.

### 3.2. Participants with SDOH

**Figure 2:**
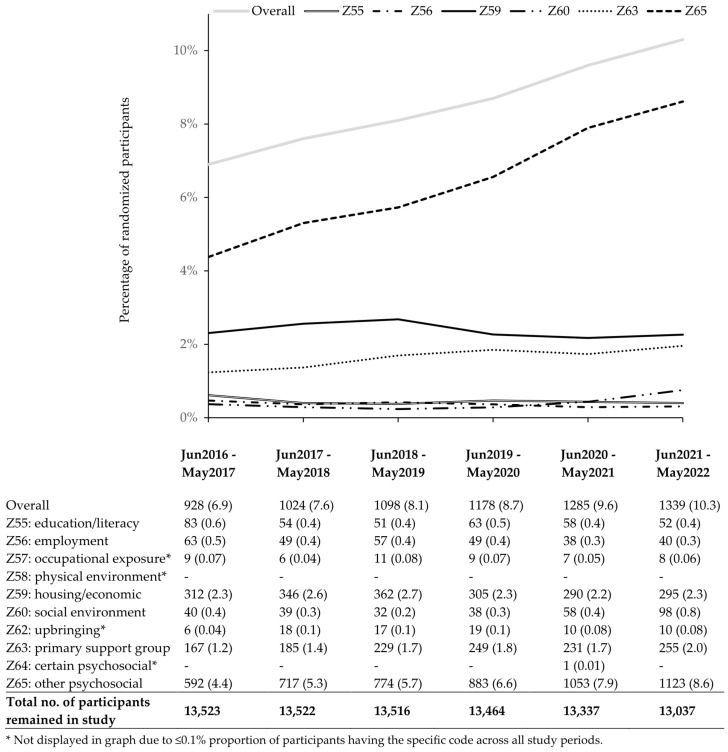
Percentage of participants with social determinants of health (SDOH) Z-code, June 2016 through May 2022.

An increased use of the SDOH codes was observed in patient-level data as well (Figure 2). 6.9% of participants had an SDOH diagnosis in the first year, and this increased to 7.6%, 8.1%, 8.7%, 9.6%, and 10.3% in each successive year. A larger percentage of participants had Z65 compared to Z59, and, notably, the percentage of participants with Z65 grew from 4.4% at study start to 8.6% by the end of the study. The proportions remained steady across other codes, with less than 1% of participants diagnosed each year for problems with education/literacy, employment, occupational exposure, social environment, and upbringing. Of the 3894 SDOH-present participants, 2286 (58.7%) had only one yearly record. The remaining 1608 (41.3%) had SDOH records for more than one separate study year. Of these, 22.2%, 10.0%, 4.6%, 2.8%, and 1.8% had Z-codes recorded in a total of 2, 3, 4, 5, and 6 years, respectively.

### 3.3. Outpatient Utilization

Social work clinics had the highest SDOH diagnosis count (Table 2), with 32.7% of all Z-codes reported originating from social care services. However, this use was most often documented as the secondary rather than the primary purpose of outpatient visits (Supplemental Tables S1 and S2). Mental health clinics were the second most frequent utilizers. VA programs providing housing care were in the 3rd position overall, but 1st when limited to the primary clinic stop codes. SDOH records were often generated via telehealth or remote visits (n = 9179; 31.3% of total records), and the vast majority of these (n = 9004; 98.1%) were generated by outpatient services whose clinic stop codes were flagged as the primary healthcare provider.

## 4. Discussion

Over the past decade, an increase in the use of SDOH diagnosis codes has been observed within both small and large US healthcare settings [12,13,24,25], including the VA, as shown in this analysis. Current findings showing an increase in ICD-10 SDOH records may not reflect improvements in screening frequency at the clinic level. However, a persistent increase in the ICD-10 SDOH records suggests a positive movement towards electronic documentation to support clinical decision-making and patient care coordination [26,27].

While recent trends in the use of the ICD-10 SDOH codes are improving, our results suggest that the improvement is minimal (increased by 3% over 6 years). SDOH code utilization appears to be less than the proportion of patients presenting SDOH risks. Our results indicated that 29% of the VA patients had an SDOH record over 6 years, while the demographic data showed 45% resided in rural areas and 69% were from lower socioeconomic backgrounds [21]. Similarly, the ACORN project found that 50% of the 268 patients reported health-related social needs through a screening initiative implemented for the New England Healthcare System in 2019 [18].

Compared to other populations, Veteran patients show a higher rate of SDOH code use. A cross-sectional analysis performed on VA patients between 2015 and 2016 (within the Veterans Integrated Service Network-4) reported that 16% of their patients had adverse SDOH documented electronically [28]. 10.3% of the DCP cohort had an SDOH ICD-10 code at study end (Figure 1). In contrast, the 2016–2017 National Inpatient Sample for US hospitalizations showed that about 6.5% of older adults had an SDOH record [12]. The 2018–2020 Medicare records for older patients with established hypertension suggested that the proportions with SDOH records were consistently below 0.5% throughout the years [25], and among all CMS beneficiaries, less than 2% had SDOH documentation in 2017 and 2020 [16,17].

The VA has a strong focus on providing equitable and high-quality care to all patients [5], and a coordinated care approach is implemented to manage the unique needs of Veterans. Eliminating homelessness is a healthcare focus for VA, and a multi-layer approach is implemented to address the problems, including the Homeless Patient Aligned Care Team (H-PACT), which offers supportive housing solutions (e.g., housing vouchers) to patients requiring assistance [29]. Adding social workers and mental health specialists to primary care teams is another VA initiative to address SDOH-related health challenges [30]. More recently, the VA began to offer Digital Divid Consults, helping patients with various healthcare-related technological obstacles [31]. These clinical interventions may enhance the overall utilization of SDOH codes, and VA patients are vulnerable to adverse SDOH, given the potential transitioning difficulties and risks for mental health problems, leading to a higher prevalence of homelessness and psychosocial issues than the general population [32,33,34].

The VA is also at the forefront of implementing risk assessments to guide patient-centered care [35]. Prior integrations include system-wide screenings to assess mental and environmental health issues, such as military sexual trauma, housing problems, food insecurity, toxic exposure, and intimate partner violence. The multidimensional socio-environmental factors, including the intricacy of co-occurring social risks, are well-recognized by VA [36]. As described, the ACORN initiative was established recently by regional healthcare providers and positioned to screen for unmet healthcare needs that can be addressed through available VA interventions [18,37]. The ACORN screening tool has nine domains to evaluate problems related to housing, utilities, transportation, education, and employment problems, as well as food insecurity, legal matters, interpersonal violence, and social isolation. However, the ICD-10 codes do not contain a mechanism to define problems for all SDOH domains that were being assessed, potentially influencing the availability of SDOH data. While 50% of VA participants from the ACORN project reported health-related social needs [18], we found that the proportion of veteran patients with ICD-10 SDOH records was relatively lower (with this study showing about 29% had SDOH records over 6 years and a previous VA study suggesting 16% based on 1-year data) [28]. Provider time constraints and inexplicit referral protocols may be other reasons for the lower use in real-world practice [38].

Implementing routine SDOH risk assessments is crucial for addressing social-related health challenges. Our results based on ICD-10 data cannot confirm how often SDOH screenings were completed in clinical environments. However, our findings suggest that these screenings might be performed less than annually, with almost all participants (>95%) having an outpatient visit during each study year, but only 41% of the SDOH-present participants had another SDOH record in subsequent years. The VA does not mandate systemwide SDOH screening, and some VA facilities may perform more frequently based on local practice (such as those that participated in the ACORN project).

Evaluating patients’ SDOH through primary care is considered the pathway for initiating routine evaluation [27,39]. While primary care clinicians alone are not sufficient to address systemwide challenges, they are the mediators who have first contact with patients prior to a major clinical event [40]. A common concern expressed by clinicians is the time required to complete all interview questions during regular visits [41,42], and efficient assessment tools are necessary for sustainable clinical implementation.

Re-screening is also important given that patients with adverse SDOH tend to have chronic diseases [25,27,43,44], and the recapture of SDOH data requires standardized screenings. Similarly to the situations with ACORN’s screening initiative, hospitals that participated in the CMS Inpatient Quality Reporting program attempted to perform SDOH screenings per the new provisions. However, there are no universal screening tools that can measure all domains of interest (food, housing, transportation, utility, and interpersonal needs) [11]. Hospitals are recommended to evaluate SDOH by combining several validated screening tests. There are SDOH screening initiatives across health systems, but universal screening tools are not yet available, suggesting a priority to establish a standardized SDOH measure for systemwide integrations.

SDOH records generated through remote visits (virtual, telephone, and home-based) accounted for about 31% of the total SDOH claims. Of these, 98% were reported by the primary service providers. There may be differences in structural or coding practice between tele- and clinic-visits, which may influence the number of SDOH records generated through secondary care for remote appointments. While health systems are starting to shift towards remote visits for patient monitoring, it is important to ensure the emerging care model offers equal awareness of SDOH and comparable data collection frameworks.

Nevertheless, the present analysis indicates that the ICD codes are being utilized across diverse patients. In our sample containing 45% rural residents and 69% low-income Veterans, Z59 and Z65 were highly coded for problems with housing/economic and psychosocial issues. Z-code documentation was higher in Medicaid beneficiaries compared to Medicare and other private insurers, with the most common SDOH as Z59 for housing or economic issues [25]. Additional guidelines may be needed for specifying situations in which Z-codes are applied. Using examples from the DCP study, a larger percentage of participants received a record of Z65 as the trial progressed, up 4.2% by the end of the study, suggesting increased problems related to “other” psychosocial circumstances. While Z65 was gaining attention, Z64, describing problems with “certain” psychosocial circumstances, was essentially unused during this time.

### Limitations

There are several limitations that may affect the generalizability of our findings. First, the DCP study cohort is older (average age = 72 years), male-dominant (97%), mostly Caucasian (77%), and all had a history of hypertension as compared to the general population [20]. Second, VA patients may have greater social care needs, leading to a higher odds of having SDOH records relative to patients in non-VA settings. Third, one of the ICD-10 SDOH codes examined, Z58 for problems related to the physical environment, was authorized for use in October 2021 (8 months before the end of our study). Fourth, current results cannot confirm whether the changes in Z-code utilization were driven by improving SDOH completion rates or worsening SDOH conditions among patients. Finally, the report on SDOH data availability cannot verify that the increased use of the ICD-10 SDOH codes was associated with improved screening.

## 5. Conclusions

The use of the structured social determinants of health (SDOH) ICD-10 codes has increased in recent years, both within the VA and other healthcare settings. The VA may have a higher level of application compared with systems providing care to the general population due to its integrated nature and having a patient population with greater social care needs. Recent VA initiatives to enhance social risk screenings and specialty clinic referrals may also facilitate SDOH documentation in EHR. Data collection of non-medical factors, such as information reflecting adverse SDOH, can be tremendously useful for advancing precision medicine and data-driven healthcare.

Standardized screening tools are needed for systematic data capture, and universal screening would likely require an organizational mandate. SDOH-related practice should be formulated to ensure feasibility for both clinic and remote visits, especially when healthcare is shifting towards remote visits and digital monitoring. Primary care can be a starting point to aid SDOH-related integrations, but collective efforts are important for sustainable change across the health system.

## Figures and Tables

**Table 1:** Patient characteristics by presence or absence of social determinants of health (SDOH) diagnosis.

	SDOH-Present (No. of Patient = 3894)	SDOH-Absent (No. of Patients = 9629)
Age, yrs—median (IQR)	71.0 (68.0–75.0)	72.0 (69.0–75.0)
BMI, kg/m^2^—median (IQR)	31.0 (27.4–35.2)	31.2 (27.9–35.0)
Systolic blood pressure, mmHg—median (IQR)	137.0 (130.0–147.0)	136.0 (129.0–146.0)
Sex		
Female—n (%)	162 (4.2)	296 (2.8)
Male—n (%)	3732 (95.8)	9360 (97.2)
Race—n (%)		
Asian	8 (0.2)	20 (0.2)
Black or African American	877 (22.5)	1150 (11.9)
Pacific Islander, American Indian, or Hawaiian	64 (1.6)	144 (1.5)
White	2711 (69.6)	7743 (80.4)
Multiple race	31 (0.8)	57 (0.6)
Unknown due to missing data	203 (5.2)	515 (5.4)
Not Hispanic or Latino—n (%)		
Hispanic or Latino	232 (6.0)	262 (2.7)
Not of Hispanic or Latino	3535 (90.8)	9014 (93.6)
Unknown due to missing data	127 (3.3)	353 (3.7)
Patient residence *—n (%)		
Rural areas	1437 (36.9)	4685 (48.7)
Urban areas	2451 (62.9)	4924 (51.1)
Unknown due to missing data	6 (0.2)	20 (0.2)
Smoking history—n (%)		
Current	1016 (27.9)	1941 (21.6)
Former	1529 (42.0)	4311 (48.0)
Never	1037 (28.5)	2449 (27.3)
Unknown due to missing data	63 (1.7)	283 (3.5)
Medical History—n (%)		
Diabetes	1843 (47.3)	4186 (43.5)
Heart failure	393 (10.1)	658 (6.8)
Myocardial infarction	198 (5.1)	290 (3.0)
Stroke	440 (11.3)	589 (6.1)
Estimated GFR < 60 mL/min/1.73 m^2^	927 (25.1)	2170 (23.9)

* Based on the VA urban/rural/highly rural (URH) classification.

**Table 2:** Outpatient service with the highest social determinants of health (SDOH) Z-code utilization.

VA Outpatient Service— Categorized by Specialized Resources		VA Outpatient Clinic Name	N (%) of 47,063 Clinic Stop Codes Associated with the SDOH Claims ^#^
Social Work (1st)			15,370 (32.7)
	125	Social work service	15,066 (32.0)
	182, 184	Care/Case manager	254 (0.5)
	680	Home and community-based service	29 (0.1)
	121	Community residential care	13 (<0.1)
	166	Chaplain Service	8 (<0.1)
Mental Health (2nd)			9018 (19.2)
	502, 527, 550	General mental health clinics	3652 (7.8)
	509, 510, 538, 576, 579	Psychiatric clinics	1314 (2.8)
	586, 587, 596, 597, 598, 599	Residential rehabilitation treatment program (RRTP)	1122 (2.4)
	535, 536, 575	Mental health vocational assistance	811 (1.7)
	568, 574	Mental health compensated work therapy	780 (1.7)
	528, 546, 552, 567, 573, 582,583,584	Intensive mental health services ^^^	676 (1.4)
	513, 514, 519, 523, 545, 547, 548, 560	Substance use disorder clinics	457 (1.0)
	516, 542, 562	Services for post-traumatic stress disorder	156 (0.3)
	533, 564, 566	Other mental health programs	50 (0.1)
Housing Services (3rd)			8487 (18.0)
	507, 522, 530	Veterans Affairs supportive housing from the US Department of Housing and Urban Development (HUD-VASH)	6844 (14.5)
	508, 529	Home care for homeless Veterans (HCHV)	1195 (2.5)
	504, 511	Grant and per diem (GPD) program	390 (0.8)
	555, 556	Homeless Veterans community employment services	58 (0.1)
Primary Care (4th)			7455 (15.8)
	147, 156, 157, 170, 172, 173, 177, 178, 338	Telephone or home-based primary care	4430 (9.4)
	301, 322, 323, 348, 531, 534, 539	Primary care, general internal medicine, and primary care mental health integration (PCMHI)	3025 (6.4)
Nursing and Other Medical Staff (5th)			1277 (2.7)
	117, 119, 171, 185, 187	Nursing	1133 (2.4)
	186	Physician assistant	62 (0.1)
	188	Fellow or resident	46 (0.1)
	209	Visit coordinator	36 (0.1)

^#^ Combining records generated through primary and secondary clinic stop codes, regardless of in-clinic or remote visits. ^^^ Included Intensive community mental health recovery (ICMHR), homeless chronically mentally ill (HCMI), and psychosocial rehabilitation and recovery centers (PRRC).

## Data Availability

The data presented in this study are available on request from the corresponding author. The datasets used or generated from the current study are not publicly available. De-identified, aggregated data may be provided upon request through an approved VA Data Use Agreement.

## Supplementary Materials

The following supporting information can be downloaded at: https://www.mdpi.com/article/10.3390/healthcare13212710/s1, Figure S1: CONSORT; Table S1: Primary Outpatient Service with the Highest Social Determinants of Health (SDOH) Z-code Utilization; Table S2: Secondary Outpatient Service with the Highest Social Determinants of Health (SDOH) Z-code Utilization.

## Abbreviations

The following abbreviations are used in this manuscript:

ACORN	Assessing Circumstances and Offering Resources for Needs
CMS	Centers of Medicare and Medicaid Services
CV	Cardiovascular
CDW	Corporate Data Warehouse
DCP	Diuretic Comparison Program
EHR	Electronic Health Record
IQR	Interquartile Range
ICD-10	International Classification of Disease, 10th Revision
SDOH	Social Determinant of Health
VA	Veterans Affairs

## Appendix A

### Social Determinants of Health (SDOH) ICD-10 Codes and Descriptions

Z55—Problems related to education and literacy
Z56—Problems related to employment and unemployment
Z57—Occupational exposure to risk factors
Z58—Problems related to physical environment
Z59—Problems related to house and economic circumstances
Z60—Problems related to social environment
Z62—Problems related to upbringing
Z63—Other problems related to primary support group, including family circumstances
Z64—Problems related to certain psychosocial circumstances
Z65—Problems related to other psychosocial circumstances
