# Supplementary material for: Social Determinants of Health ICD-10 Code Use by a Large Integrated Healthcare System
Source: Healthcare (Basel). 2025 Oct 27;13(21):2710. doi: 10.3390/healthcare13212710 (PMC12608873; doi:10.3390/healthcare13212710)
Supplement: Supplementary file 1 [file healthcare-13-02710-s001.zip › healthcare-3871779-supplementary.pdf]

Figure S1. CONSORT

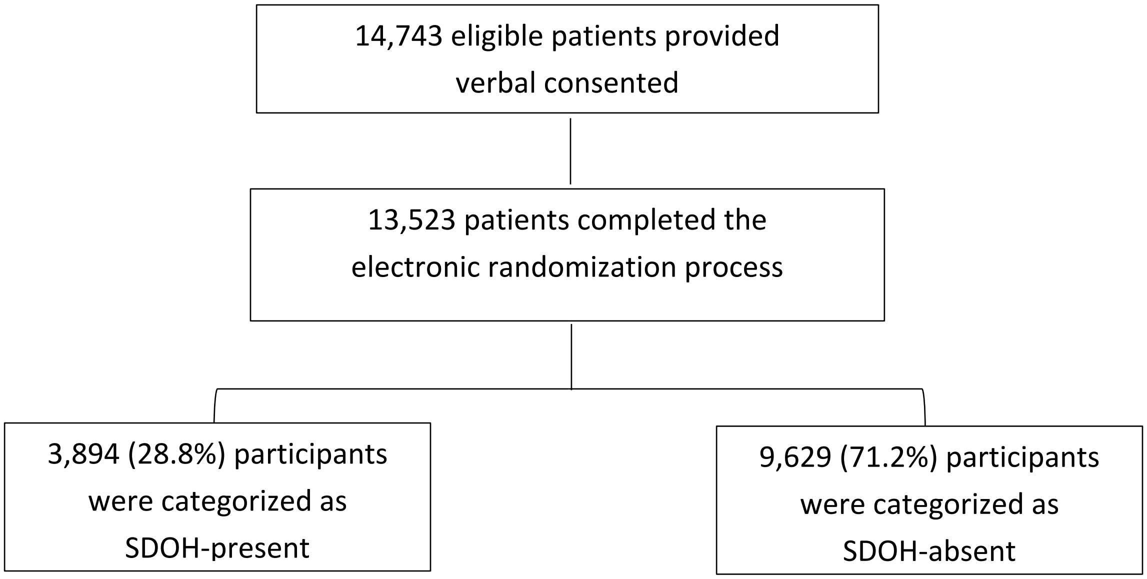

Table S1. Primary Outpatient Service with the Highest Social Determinants of Health (SDOH) Z-code Utilization

VA outpatient service – categorized by specialized resources	VA outpatient clinic name	N (%) of 27,871 primary clinic stop codes associated with the SDOH claims#
Housing Services (1st)		8,079 (29.0)
507, 522	Housing and Urban Development – Veterans Affairs supportive housing (HUD-VASH)	4,006 (14.4)
530*	Housing and Urban Development – Veterans Affairs supportive housing (HUD-VASH), telephone	2,660 (9.5)
508, 529	Home care for homeless veterans (HCHV)	1,045 (3.7)
504, 511	Grant and per diem (GPD) program	326 (1.2)
555, 556	Homeless Veterans community employment services	42 (0.2)
Mental Health (2nd)		7,023 (25.2)
502, 550	General mental health clinics	2,532 (9.1)
527*	General mental health clinics, telephone	888 (3.2)
538, 576	Psychiatric clinics	25 (0.1)
579*	Psychiatric clinics, telephone	10 (<0.1)
586, 587, 596, 598, 599	Residential rehabilitation treatment program (RRTP)	1,095 (3.9)
597*	Residential rehabilitation treatment program (RRTP), telephone	10 (<0.1)
535, 575	Mental health vocational assistance	393 (1.4)
536*	Mental health vocational assistance, telephone	278 (1.0)
568, 574	Mental health compensated work therapy	714 (2.6)
552, 567, 573, 582, 583	Intensive mental health services^	41 (0.1)
528*	HCMI^, telephone	625 (2.2)
546*	ICMHR^, telephone	9 (<0.1)
584*	PRRC^, telephone	1 (<0.1)
513, 547, 560, 548	Substance use disorder clinics	194 (0.7)
545*	Substance use disorder clinics, telephone	15 (0.1)
516, 562	Services for post-traumatic stress disorder	144 (0.5)
542*	Services for post-traumatic stress disorder, telephone	8 (<0.1)
533, 564, 566	Other mental health programs	41 (0.1)
Primary Care (3rd)		6,800 (24.4)
147*, 156*, 157*, 170*, 172*, 173*, 177*, 178*, 338*	Telephone or home-based primary care	4,430 (15.9)
301, 322, 323, 348, 534, 539	Primary care, general internal medicine, and primary care mental health integration (PCMHI)	2,370 (8.5)
Social Work (4th)		3,498 (12.6)
125	Social work service	3,408 (12.2)
182*	Case management, telephone	65 (0.2)
121	Community residential care	12 (<0.1)
166	Chaplain Service	8 (<0.1)
680*	Home and community-based service	5 (0.1)
Nursing and Other Medical Staff (5th)		119 (0.4)
119, 171	Nursing	83 (0.3)

209	Visit coordinator	36 (0.1)

#Including records submitted through primary clinic stop codes; *Notation for remote visits.

^Contained records generated by the Intensive community mental health recovery (ICMHR), homeless chronically mentally ill (HCMI), and psychosocial rehabilitation and recovery centers (PRRC).

Table S2. Secondary Outpatient Service with the Highest Social Determinants of Health (SDOH) Z-code Utilization

VA outpatient service – categorized by specialized resources	VA outpatient clinic name	N (%) of 19,192 secondary clinic stop codes associated with the SDOH claims*
Social Work (1st)		11,872 (61.9)
125	Social work service	11,658 (60.7)
184	Case management	189 (1.0)
680*	Home and community-based service	24 (0.1)
121*	Community residential care	1 (<0.1)
Mental Health (2nd)		1,995 (10.4)
502, 550	General mental health clinics	232 (1.2)
509, 510, 538	Psychiatric clinics	1,279 (6.7)
587, 596	Residential rehabilitation treatment program (RRTP)	17 (0.1)
535, 575	Mental health vocational assistance	140 (0.7)
568, 574	Mental health compensated work therapy	66 (0.3)
513, 514, 560, 519	Substance use disorder clinics	248 (1.3)
562	Services for post-traumatic stress disorder	4 (<0.1)
533, 564	Other mental health programs	9 (<0.1)
Nursing and Other Medical Staff (3rd)		1,158 (6.0)
117, 119, 185, 187	Nursing	1,050 (5.5)
186	Physician assistant	62 (0.3)
188	Fellow or resident	46 (0.2)
Primary Care (4th)		655 (3.4)
301, 322, 323, 531, 534	Primary care, general internal medicine, and primary care mental health integration (PCMHI)	655 (3.4)
Housing Services (5th)		408 (2.1)
507, 522	Housing and Urban Development – Veterans Affairs supportive housing (HUD-VASH)	178 (0.9)
508, 529*	Home care for homeless veterans (HCHV)	150 (0.8)
511	Individual grant and per diem (GPD) program	64 (0.3)
555	Homeless Veterans community employment services	16 (0.1)

Including records submitted through secondary clinic stop codes; *Notation for remote visits.
